# The 4Cs of Housing: Resident Perspectives on Cost, Conditions, Consistency, and Context in a Public Housing Neighborhood

**DOI:** 10.1080/10511482.2026.2669261

**Published:** 2026-05-29

**Authors:** Samantha Teixeira, Zhirui Chen, Rebekah Levine Coley

**Affiliations:** a Boston College School of Social Work, Chestnut Hill, MA, USA;; b Boston College Lynch School of Education and Human Development, Chestnut Hill, MA, USA

**Keywords:** Public housing, development/revitalization, neighborhood, quality, rental housing, urban environment

## Abstract

The residential environment, encompassing housing and neighborhood of residence, is a critical context shaping health and well-being. The 4Cs model delineates four pathways through which residential environments affect health including cost, conditions, consistency, and context, all of which are often compromised for low-income individuals. Affordable housing programs, like public housing, should mitigate negative exposures across the 4Cs but disinvestment has left many public housing developments with maintenance deficiencies and disadvantaged contexts. Through analysis of qualitative interviews with 61 residents of a large urban public housing development, we elucidate residents’ perspectives of the ways the 4Cs shape experiences and well-being in contemporary public housing. Findings suggest that the 4Cs are interrelated and mutually reinforcing. Participants endured poor conditions and viewed them as a necessary trade-off to maintain consistency, because moves are cost prohibitive and may require moving outside of their neighborhood, losing the amenities and kinship they valued in the context. Impending redevelopment increased fears of disrupted consistency, overshadowing hoped-for improvements in context and conditions. These findings hold important policy implications, as such trade-offs may undermine the goals of the public housing program to provide decent, stable, affordable housing.

## Introduction

The residential environment, encompassing both housing and neighborhood of residence, is a critical context shaping health and well-being (Chyn & Katz, 2021; Devoss et al., 2022; Swope & Hernández, 2019). Economically and socially marginalized individuals, particularly renters, face shortages of affordable, high-quality housing and increasingly economically segregated communities, particularly in gentrifying US cities. Low-income renters are more likely than their more advantaged peers to live in residential contexts marked by disorder and precarity, in which risks to health such as material hardship, exposure to physical hazards like mold and lead, and overcrowding and social disorder exacerbate risks for negative outcomes and contribute to broad disparities in health and well-being (Adamkiewicz et al., 2011; DeLuca & Rosen, 2022).

Understanding the risks to health and well-being presented by a lack of access to safe, affordable housing and communities, government and private providers have invested significant resources into housing assistance for low-income households. The US federal government supports low-income households through three primary programs: tenant-based rental assistance (vouchers), project-based rental assistance, and public housing (Aratani et al., 2019). Public housing, the longest-running model of government housing assistance, currently serves approximately 970,000 US households, with an $8.9 billion federal investment (HUD, 2024). Public housing seeks to “provide decent and safe rental housing for eligible low-income families, the elderly, and persons with disabilities” at affordable prices (HUD, 2024) and, as such, seeks to promote affordable costs, decent conditions, consistency, and a safe context. Yet mixed evidence has emerged. Public housing caps housing costs and lowers costs in response to a loss in income, providing affordability and consistency. In fact, some research suggests that public housing may provide long-term benefits compared to other low-income rental situations by providing consistent, affordable housing (Ruel et al., 2010). There are reasons to believe that the benefits of affordability may be growing. In the current financial context in which inadequate new housing development and a rapid and explosive growth in housing prices have dramatically decreased the availability of affordable options for low-income renters, public and subsidized housing options have become ever more financially precious (Airgood-Obrycki et al., 2024). Such shifts may alter how residents view the benefits of public housing.

However, these strengths of public housing may be counterbalanced by housing and neighborhood contexts marked by poor conditions. Many contemporary public housing developments have experienced decades of underinvestment, leading to severe maintenance deficiencies. The National Association of Housing and Redevelopment Officials (NAHRO) estimates that $90 billion in backlogged repairs are needed in the nation’s public housing, yet efforts to fund needed improvements, including the Public Housing Emergency Response Act (2024) introduced in 2021 and reintroduced in 2024, have stalled in Congress. These profound unmet capital needs leave public housing residents facing compound disadvantages across conditions and contexts of their housing, with a lack of resources to buffer these risks. Large-scale efforts in the 1990s and 2000s, namely the federal HOPE VI program, aimed to rebuild the most severely distressed public housing developments and improve community resources and contexts. This program succeeded in creating some positive contextual changes for communities in terms of lowering concentrated poverty and improving the housing stock (Coley et al., 2023; Teixeira et al., 2026), but dramatically interrupted consistency through large-scale displacement leading to declining housing and neighborhood conditions for many residents (Goetz, 2011). Moreover, HOPE VI, and the newer Choice Neighborhoods program, only targeted a very small proportion of public housing developments, leaving most continuing with notable challenges. For example, a 2019 estimate suggested that only 22% of public housing properties have undergone construction in the past 30 years and 42% completed their most recent construction before 1975 (Docter & Galvez, 2020).

The demographic and economic makeup of public housing and surrounding communities have shifted as well. Through the second half of the 20^th^ century, public housing transitioned from a context for working-class White families to increasingly serving high-poverty and racially segregated communities of color (Rothstein, 2017). Following postwar suburban development and White flight, the neighborhoods surrounding public housing similarly grew in the concentration of poverty and racial segregation, isolating residents from employment opportunities and resources and entrenching social disorder (Nebbitt, 2015; Popkin et al., 2009; Rothstein, 2017). Extensive research, much conducted in a handful of large US cities such as Chicago and New York, has documented the growth of urban poverty and social isolation, particularly among Black residents, with influential books such as *The Truly Disadvantaged* (Wilson, 1987) expanding conceptualizations of urban decay. Although civil rights policies and legal cases led to small declines in racial segregation in public housing in the late 20^th^ century (Goering et al., 1994), limited research has assessed integrated communities. In recent years, however, with public housing redevelopment, urban gentrification, and the growth in higher socioeconomic status (SES) households opting to live in racially and ethnically diverse urban neighborhoods, many public housing developments are now surrounded by more racially and economically contrasting environments (Ellen & Torrats-Espinosa, 2019), with a need for updated evidence assessing residents’ contexts and experiences. Such environments could, for example, provide greater economic and social opportunities for public housing residents or, alternately, could increase their exposure to economic or racial discrimination and social isolation as local services become more expensive and exclusive (Chaskin & Joseph, 2019; Chetty et al., 2026).

Although numerous investigations have sought to understand the effects of both place-based redevelopment policies (e.g., Coley et al., 2023; Teixeira et al., 2026) and person-based relocation policies (see Chyn & Katz, 2021 for a review), it is important to understand the residential contexts and experiences of the thousands of public housing residents who have not been the recipients of these opportunities and who face changing urban contexts combined with growing repercussions of public housing disinvestment and shrinking affordable options in the private housing market. To address this need, we draw on Swope and Hernández’s (2019) conceptual model identifying four pillars of housing—*cost*, *conditions*, *consistency*, and *context*—which serve as pathways by which residential environments affect health. Building upon this framework, which we call *the 4Cs of housing*, we seek to deeply explore current public housing residents’ experiences of their residential environment, asking:

- What are public housing residents’ perceptions and experiences of each of the 4Cs (cost, conditions, consistency, and context), and how do the 4Cs intersect?
- How salient are each of the 4Cs to their residential experiences and well-being?

We locate this assessment within a conceptually and politically important context: a racially and ethnically diverse urban public housing development, situated in a resourced, rapidly gentrifying and high housing cost community. Through analysis of qualitative interviews with 61 adult residents, we seek to expand understanding of residents’ experiences with each of the 4Cs, and better understand how the 4Cs act individually and interdependently to shape residents’ experiences and well-being. Through these empirical goals, we seek to better integrate and hence strengthen the conceptualization of the 4Cs framework and identify implications for policy and future research.

## Background: The 4Cs and Public Housing Contexts

The 4Cs conceptual model identifies four key aspects of residential environments: *cost*, *conditions*, *consistency*, and *context* (Swope & Hernández, 2019). Public housing is an interesting context for empirically elucidating the individual dimensions and intersections of the 4Cs model because many elements of the U.S. public housing program’s design aim to minimize risks across these four domains. DeVoss et al.’s (2022) systematic review delineated several ways in which subsidized housing relates to the 4Cs. Here, we expand upon the proposed linkages and deepen the focus on public housing communities, in particular.

*Cost* represents housing affordability, or residents’ ability to pay for housing without burden (Swope & Hernández, 2019). The *cost* of federal public housing rental units is determined by a formula in which the total tenant payment, including utility allowances, is generally set at 30% of a household’s adjusted income, as it is at our study site (Boston Housing Authority, 2025a). These cost protections have been found to help maintain affordability and limit cost-saving behaviors, such as doubling up, limiting energy usage, and relying on food assistance, which are commonly undertaken among other low-income renters (Aratani et al., 2019; Rosen et al., 2023). Such limits in rental costs may also benefit health and well-being. For example, doubling up and overcrowding may ease rental burdens but have been associated with adverse health outcomes including poorer mental and behavioral health among adults and children, as well as physical health problems (Swope & Hernández, 2019). Other cost-cutting strategies such as reducing utility usage have been associated with negative outcomes including psychological distress and adverse physical health outcomes (Hernández, 2016; Turney et al., 2013). The cost protections built into public housing may be growing in import as housing demand outstrips supply and housing prices increase far faster than the rate of inflation (Myers et al., 2023). However, public housing residents may also incur stress related to these variable rental costs, as rent automatically increases following increases in income, and growing disparities between rental costs in subsidized versus private market housing may lead public housing residents to feel stuck in place, even in the face of stress from other characteristics of their housing. DeVoss et al. (2022) identified the need for increased understanding of how public housing tenant rent calculation policies intersect with employment and other economic decisions and their relationship to mental health.

*Conditions* are physical and structural aspects of housing that may impede health, including factors such as deterioration and deferred maintenance, inadequate temperature control, pests, and environmental insults or unsafe conditions (Swope & Hernández, 2019). *Conditions* in public housing are governed by local and federal regulations related to sanitation and the prevention of substandard housing. Yet despite these mandates, public housing residents frequently experience deficient housing conditions (Lemire et al., 2022; Popkin et al., 2009). While some research suggests that public housing residents experience better conditions than low-income counterparts in other subsidized rentals, other rigorous research has found worse conditions (Van Ryzin & Kamber, 2002) or few differences in housing quality (Lemire et al., 2022; Mast, 2012). In Boston, where this study takes place, decades of disinvestment have left the city’s public housing deeply under-resourced, with a recent report estimating $1.5 billion in deferred capital needs and an inability to respond promptly to pressing resident concerns regarding housing conditions (Kummerer, 2024). While significant research has documented neglected, substandard housing conditions in public housing (e.g., Popkin et al., 2009) there remains a need to better understand housing conditions in contemporary public housing.

This remains an important area of research because deficient housing conditions have been associated with a host of deleterious health and mental health outcomes in a robust body of research. For example, conditions such as pests, dampness and mold, secondhand smoke, and pollutants in indoor air are more prevalent in the homes of low-income renters and have been associated with poorer respiratory health and higher exposure to allergens and chemicals (e.g., pesticides) (Adamkiewicz et al., 2011; Swope & Hernández, 2019). A number of studies have examined the relationship between deficient housing conditions in public housing and their relationship to mental health, though most of these focus on experiences following relocation or renovation, with less attention to the experiences of sustained exposure to housing deficiencies in contemporary public housing (DeVoss et al., 2022; Schmidt, 2024). Some research has pointed to the survival strategies that low-income renters employ in response to poor conditions (Hernández, 2016) and the psychosocial toll of deficient conditions, including feelings of powerlessness, anxiety, and stress, pointing to a need to assess how sustained exposure to such conditions may relate to resident well-being, and may intersect with other aspects of the 4Cs (DeVoss et al., 2022; Schmidt, 2024).

*Consistency* is defined in the 4Cs framework as residential stability, with stability encompassing a resident’s ability to remain in her home and the absence of threats of homelessness and displacement, overcrowding, and serial or forced moves. Housing consistency among public housing residents has received less attention in the literature, perhaps because public housing is thought to offer the most consistency and affordability of the subsidized housing arrangements in the U.S (Leung et al., 2023). Rather, studies of public housing focused on consistency have most commonly assessed experiences of forced displacement as a result of redevelopment policies such as HOPE VI. These studies have identified numerous challenges associated with displacement, including disrupted social connections as well as worsening conditions and increasing costs in housing (Keene & Geronimus, 2011). Substantially less research has focused on experiences of residential stability among low-income and subsidized renters, and how much of such stability is desired versus enforced through pressures from rising unaffordability in other housing options (Schmidt, 2024).

Public housing residents may stay in public housing as long as they comply with the lease (with costs lowering in response to a loss of income), which may offer substantial stability in comparison to private or voucher housing. Overall, the existing research has found that housing assistance, and public housing in particular, promotes consistency, and that stability (operationalized as fewer moves and longer tenure) is associated with better mental health, whereas displacement and moves are associated with poorer mental health across studies (DeVoss et al., 2022). Yet recent research suggests that in the face of austerity and pressure to collect rent, public housing authorities use similar strategies to private market landlords, including serial eviction filings and court processes to facilitate rent collection (Leung et al., 2023). Indeed, Leung and colleagues (2023) found in a national study that public housing residents were at equal risk for eviction filings as private market neighbors, and faced significantly higher serial eviction filings, representing notable risks to consistency.

Large-scale displacement of public housing residents also occurred during earlier redevelopment efforts, with evidence suggesting negative effects on residents (Keene & Geronimus, 2011; Popkin et al., 2009). In response, newer models are implementing phased redevelopment plans which seek to limit the proportion of residents displaced as well as the length of displacement, and often include greater communication and community engagement in redevelopment planning (Chaskin & Joseph, 2019). Yet, such redevelopment models may introduce new challenges due to extended redevelopment timelines and the experience of remaining in place in deficient housing units, with the surrounding community undergoing active demolition. Limited evidence exploring residents’ experiences as they remain in place awaiting public housing redevelopment have highlighted “waiting” as a potent psychological stressor and threat to *consistency* (Watt, 2023; Westbrook et al., 2024), but further research is needed to understand the potential tradeoffs of such redevelopment models.

Finally, *context* refers to the broader community context in which public housing is located, including neighborhood characteristics and resources such as institutions, social characteristics, walkability and transit access, and safety (Swope & Hernández, 2019). National data indicate notable increases in income inequality (Guzman, 2022) as well as in the spatial concentration of both poverty and wealth, leading to increased economic isolation in many poor neighborhoods (Bischoff & Reardon, 2014). Public housing communities often exemplify these patterns, being located in underinvested communities isolated from social and economic opportunities (Coley et al., 2023; Nebbitt, 2015). A substantial literature identifies high levels of neighborhood violence and a lack of resources in the neighborhood environments of public housing developments (DeLuca & Rosen, 2022; Popkin et al., 2009), though these safety concerns may be buffered by supportive kinship networks and social cohesion (DeVoss et al., 2022). Less evidence has addressed other key aspects of context, such as access to greenspace and facilities such as public transportation, recreational opportunities, and commercial services (Coley et al., 2023; DeVoss et al., 2022). Rigorous research has established nuanced links between neighborhood features and health and well-being, finding that both adults and children who move from disadvantaged neighborhood environments to neighborhoods with fewer environmental stressors (e.g., pollution, violence, lack of resources) experience enhanced health outcomes and, for children, improved long-term well-being (Chyn & Katz, 2021). Some single-site and qualitative studies have found that public housing residents report higher levels of social cohesion than voucher-holding counterparts (Donley & Nicholson, 2019), and that this positively influences mental health and may buffer the negative influence of contextual stressors (DeVoss et al., 2022).

Taken together, policies and practices governing public housing suggest numerous areas of protection in the 4Cs. The extant research, however, is more mixed, suggesting that while subsidized housing, and public housing in particular, may offer *consistency* and *cost* controls as compared to other forms of rental housing, it may also exert unique stressors in the domains of *conditions* and *context*. Moreover, changing contexts—driven by rising housing costs, increasing racial diversity and neighborhood gentrification, and continued public housing redevelopment—may have altered the experiences and intersections of the 4Cs among contemporary public housing residents. In this work, we seek to add to the literature in three primary arenas. First, we provide a rich exploration of each of the 4Cs in a racially and ethnically diverse sample of public housing residents living in one of the oldest and hence most deteriorated public housing sites in New England, but also a community located within a gentrifying and well-resourced broader neighborhood. Second, we seek to deeply explore potential tradeoffs between components of the 4Cs from the perspectives of public housing residents themselves. Third, by studying a public housing community slated for redevelopment under the new model of limited displacement and greater community involvement, we seek to provide early evidence on how this process is perceived and experienced by residents. Through this effort, we seek to further elucidate the 4Cs model, to expand empirical evidence of the housing experiences of low-income renters in contemporary public housing, and to provide conceptual and policy insights.

## Data and Methods

This manuscript draws on in-depth, semi-structured interviews with 61 adult residents of a large, urban public housing development. The interview data were collected as part of the Housing Opportunity and Mobility Experiment (HOME) Study, a longitudinal, mixed-methods study of residents of the oldest and one of the largest public housing developments in New England, originally built in the 1930s, which is slated for redevelopment into a mixed-income community. The development includes approximately 1,000 households (54% families), and approximately 2,000 residents living in 3–4 story apartment buildings with shared entrances or in attached two-story townhomes with private entrances and shared courtyards. The development contains notable diversity along racial/ethnic (13% Asian, 27% Black, 38% Hispanic, and 22% White residents) and age strata (29% children; 10% young adults, age 18–24; 44% working-age adults, age 25–61 years; and 18% older adults, age 62+). It is home to many long-term residents, with an average tenure of 16 years among adult residents, and is surrounded by a much less racially diverse neighborhood that is rich with amenities including parks and beaches, public transit, and an array of commercial services.

Data for this study were drawn from the first wave of the HOME Study. At the time of data collection, residents had been informed that their neighborhood was slated for redevelopment through numerous community meetings and fliers seeking to inform and garner feedback from residents, but relocation, demolition, and construction had not yet been initiated. The phased redevelopment process aims to limit external displacement, with most residents moving directly into newly built units while approximately 10–20% of households will be temporarily displaced off site then returned during the redevelopment process, with all current residents guaranteed a unit in the redeveloped community. Over approximately 10 years, the redevelopment process will result in a much more populous mixed-income development, with approximately 1/3 affordable and 2/3 market rate units.

The HOME Study includes surveys with adults and youth (aged 12–24), environmental monitoring, and biological samples, as well as semi-structured interviews and collation of administrative data. The survey sampling plan targeted 664 households (including households targeted for initial stages of redevelopment and demographically matched comparison households), with recruitment conducted through mailings followed by face-to-face and electronic contacts. Surveys were conducted in person and online in English, Spanish, Cantonese, and Mandarin from October 2022 through January 2024, with 73% of eligible households participating. From the survey sample, we selected a subsample of households to participate in the semi-structured interviews, stratified by redevelopment stage, household type, and race/ethnicity.

A team of interviewers, including the first author of this manuscript, conducted the semi-structured interviews in English, Spanish, Cantonese, and Mandarin with 50 adults and 25 youth aged 12–24 (together representing 55 unique households) between August 2023 and March 2024, with 89% of eligible households participating. We matched interviewers to participants based on language and, where possible, race and ethnicity. Interviewers used a semi-structured protocol focused on perceptions of the neighborhood and interactions with neighbors, housing-related issues, parenting and family life, school and work, and health and mental health. We conducted interviews in person in participants’ homes or in a private community space, with the exception of one interview that was conducted via Zoom at the participant’s request. Participants were compensated with a gift card ($60 for household heads; $40 for other household members). Interviews averaged 55 minutes long and were audio recorded and transcribed verbatim. Spanish and Chinese transcripts were then translated into English for analysis. All procedures were approved by the Boston College Institutional Review Board.

### Sample

This analysis focused on the 61 interview participants aged 18 and above from 54 households. Table 1 summarizes sample characteristics. Participants ranged in age from 18 to 92, with a mean age of 46 years. The sample was racially and ethnically diverse, with the largest group identifying as Hispanic (39%), followed by Non-Hispanic White (20%), Non-Hispanic Black (16%), Asian (15%), and multiracial (10%). Around 66% of the interviews were conducted in English, 25% in Spanish, and 10% in either Cantonese or Mandarin. Nearly two-thirds of participants lived in apartment buildings (64%), while 36% resided in townhomes. The length of residence among participants averaged 16 years, with a broad range of less than 1 year to 46 years.

### Data Analysis

We used Braun and Clarke’s (2021) Reflexive Thematic Analysis approach to analyze the interview transcripts. This approach is well suited for this study due to its utility for identifying and interrogating patterns in qualitative data, producing layperson-friendly results, and informing policy development (Braun & Clarke, 2006). We began by *familiarizing* ourselves with the data, listening to recordings of interviews and re-reading interview transcripts. We wrote memos to capture early insights which were used to develop the codebook. We then conducted a multi-cycle *coding* process using NVivo 14 software and Microsoft Excel. Coding began deductively from the interview guide and then a second cycle of coding was aligned to the 4Cs concepts such that the conceptual framework guided theme development and interpretation (Braun & Clarke, 2021). A diverse research team representing the linguistic and cultural diversity of the participants coded the transcripts, each of which was coded by two coders. In Reflexive Thematic Analysis, though a multi-coder process is not essential, when employed, the purpose is to collaborate to gain insights from the data, not necessarily to achieve inter-coder agreement (Braun & Clarke, 2021). Therefore, using a consensus-based approach, coding pairs met weekly to discuss the transcripts they had coded, identifying, discussing, and, when necessary, consulting with the lead author and other team members on all discrepancies in coding across the pair members in order to come to consensus on all codes applied to each transcript.

Next, we *generated initial themes* by sorting the coded data into a spreadsheet delineating participant descriptions of each of the 4Cs constructs. We used memo writing and ongoing review of the literature to develop the initial themes in each of the 4Cs and then *reviewed*, *defined*, and *named the themes* by assessing the fit of candidate themes with the data, again reviewing transcripts to ensure that the themes represented a pattern of shared meaning. Finally, we *wrote the results* of the analysis, which included writing vivid descriptions with illustrative examples to logically tell the story of the data. We present participant quotes verbatim where possible, though some quotes were edited in minor ways for readability (e.g., removing uh, like, and other filler words; removing tangential portions of quotes) (Lingard, 2019). In such cases, we strived to maintain the authenticity of the participants’ stories and used brackets to reflect where quotes were revised for clarity or masking purposes.

### Positionality and Reflexivity

Our authorship team comprises two White women and one Asian woman. Members of the authorship team have experience living and working in subsidized housing and collectively have decades of professional experience working with communities of concentrated poverty and public housing communities. Throughout the research process, we reflected on how our personal and professional identities might influence our interpretation of the data. Each member of the coding team wrote a positionality statement and participated in extensive group discussion about how their experiences may produce unique insights and/or lead to areas of weakness. We checked the trustworthiness and rigor of our analysis by using a multi-coder data analysis approach and peer debriefing, in which any coding discrepancies were discussed as a group. We have been working in the study community for eight years and have strong relationships with community members, including the HOME Study community advisory board, composed of residents and agency representatives who provide ongoing feedback. Members of the community advisory board have, for example, provided input into the development of survey and interview protocols, participated in pilot testing of instruments, and have reviewed baseline findings prior to disseminating more broadly to the community and professional outlets.

## Results

The results of our analysis provided insights into participants’ perceptions of *cost*, *consistency*, *conditions*, and *context* in their public housing community. We begin by delineating participant perceptions of each element of the 4Cs model. Next, we review perceptions of connections with well-being and crosscutting themes.

### Cost

In the 4Cs framework, *cost* represents housing affordability, or residents’ ability to pay for housing without burden (Swope & Hernández, 2019). As a federal public housing development, rent at the study site is set at 30% of adjusted income. Income is adjusted to take into account medical expenses that exceed 3% of household total income, childcare expenses for children under age 13, and income from household members who are full time students aged 18 and up. The housing authority also notes that residents can work with the property manager to create an alternate payment plan in the event of hardship, and partners with city and state agencies to assist tenants in accessing rent relief programs (BHA, 2025a). Utilities including heat, electricity, and water are paid by the housing authority. Despite these affordances, the majority of participants described cost-related stressors including difficulty making ends meet, inability to afford housing outside of the public housing development, and challenges related to the housing authority’s rent calculation formula that they described as limiting their upward mobility to higher quality, affordable housing.

When asked about family stressors, most participants discussed financial strain, often reporting housing-related costs to be a primary stressor. They described costs as anxiety-provoking and overwhelming. For example, one participant, a Hispanic woman in her thirties, said, “my only headache is rent…it’s only just building up. You know? And when it builds up and I don’t have it, I’m gonna be like a freak, how am I gonna get it?” She went on to describe how she had been in arrears and the only way she had been able to catch up was when a relative unexpectedly passed away and left her a few hundred dollars, which she applied to her rent. But, after paying off this debt, she had quickly fallen behind again.

Numerous participants described fears that, although public housing policy allows for adjustments related to job loss or hardship, they might lose their housing (i.e., lose *consistency*) if they reached out for assistance. For example, a White older adult man described how a job loss made him fear that he would be thrown out, so he resorted to making ends meet how he had when he lived on the streets:

When I was losing that job, I was kind of worried like, they’re not gonna turn you out? [They] get 30%. I’m like, yeah, I have no income. What’s 30% of zero? <laugh>…so, I picked up cans and I survived. I picked up cans…[and did some] panhandling.

Another resident, a young, Hispanic mother, described the overwhelming feeling that there was no way out of her financial trouble:

I do get a lot of breakdowns with, especially in the holidays. You want to buy everything your kids want. You want to give your loved ones what they want. And sometimes your budget is just not budgeting…sometimes with like the bills, all the stuff you have to pay…it’s just a lot. It’s overwhelming because it’s not something that you can ask for help. Like, I can’t call [the housing authority] and say, ‘Hey can you guys help me pay my rent?’…life is so hard, everything is so expensive. Groceries, everything’s so expensive. So, it’s overwhelming.

The feelings of stress surrounding housing costs were further complicated by the fact that participants reported feeling “stuck” and helpless to alleviate the pressures. For example, many identified intersections between *cost* and *conditions* and *cost* and *consistency*, reporting that though the cost of their rent is disproportionate to the quality of the housing they receive, they lack other affordable options. This feeling of restricted options was driven both by the lack of local affordable housing options and by perceptions that the rent calculation formula limited their ability to save to move to a higher quality home. Many residents noted a lack of affordable housing in the area and described the neighborhood surrounding the projects as gentrifying, noting the difference between the public housing development and other sections of the neighborhood as “night and day.” One woman, an Asian older adult, appreciated that the rent was capped at 30% of her income but said, “I pay $1,400 now…but, if I don’t live here, I cannot afford to live in Boston. You see how around me, all those [houses are] expensive, right?” She went on to say that she loves the city and would simply not be able to afford an apartment elsewhere, so she puts up with building conditions that she described as deteriorating with little help from management despite the high rent payment.

In addition to limited affordable housing options, participants also expressed feelings of being “trapped” by the rental calculation formula. Despite the housing authority’s pledge to support residents facing hardships, a number of participants relayed how as they increased their work hours or their children entered the labor market, their increased household income led to increases in rent, limiting their ability to save and advance. One young White woman described the stress:

I feel like [the housing authority] holds people back from doing a lot…. you can’t even, like, try to do better for yourself because if you make a little bit more, they take more… so, like, our rent is almost…like two grand, which I feel like is a little crazy if this is supposed to be affordable housing…But it’s like, you can’t get out. Everything is so much more expensive anywhere else.

Taken together, participants reported feeling stressed, angry, and overwhelmed by *cost*-related concerns, which often intersected with housing *conditions*, *consistency*, and *context*. However, their concerns were even more expansive in relation to their housing *conditions*.

### Conditions

Swope and Hernández (2019) described housing *conditions* as physical and structural aspects of housing quality that may impede health, including factors such as deteriorating conditions and deferred maintenance, inadequate temperature control, pests, and environmental insults or unsafe conditions in the broader building. Participants described housing conditions at length, with wide-ranging complaints concerning broken or malfunctioning appliances, pests and rodents within their units, and unsafe and unsanitary conditions in building common spaces. Many residents noted that conditions had worsened over time, and highlighted a lack of responsiveness by the housing authority. Some turned to doing their own maintenance, or paying for repairs out of pocket. Far fewer participants highlighted positive conditions or expressed a feeling that conditions had shown improvement, with positive views appearing more prevalent among townhouse residents.

A heavy focus of concerns regarding housing conditions related to deteriorated structural conditions and malfunctioning appliances, with residents describing their apartments as “dirty,” “unkept,” “disgusting,” and even “houses of terror,” which are old and run-down, with an array of challenges including holes in walls and peeling paint, sharp broken floor tiles, broken windows, doors that do not properly shut or lock, and malfunctioning stoves and refrigerators, conditions which at times presented notable health risks. For example, one participant, an older Black woman, reported:

I don’t have a stove. I, I’ve got a stove, but it shocked my nurse, and it shocked me [gave her an electric shock]…touched it and it just blackened my hand. And my nurse, what she did, she was leaning against the stove- and it shocked her and put black marks down her back.

Another participant, a younger Black woman, listed a longer array of challenges:

The walls peel, the refrigerator goes in and out, the kitchen light’s broke…. My stove broke, and it took them over three weeks to fix it, and we had no stove…. When you plugged it in and you turned it on, it would start sparking. Then they brought a new one out and the same thing kept happening…. We had problems with the shower. When you turn the shower on, the darn thing would just come off. It took them almost a month to fix that, to the point where we didn’t have any water, and we actually went almost six days with no shower because the water wouldn’t turn on.

Numerous other residents similarly highlighted challenges with both air and water temperature control. Residents noted the inconsistency in hot water, exemplified by this quote from a middle-aged Asian woman who said the water “often fails to work. The hot water is sometimes too hot … and it fluctuates between hot and cold.” Others described a lack of adequate heat and air conditioning, as this young Asian woman reported:

It’s stressful, the heat. Like, sometimes it’ll be like, right now it’s so hot. And so, I’ll open the doors. And I’ll lock the outside, the screen door…but the door’s open and so like the people walking by can see inside the house and see what I’m doing and things like that. And I get like, really paranoid.

As the second example indicates, issues with thermal control intersect with the broader *context* and concerns about neighborhood safety, leaving residents feeling unsafe and unable to address their discomfort.

Deferred maintenance and slow or incompetent responses to maintenance requests were also common themes, with many residents expressing a belief that the housing authority had purposely stopped responding to maintenance requests due to the upcoming redevelopment of the community. As one older Asian woman relayed when discussing the housing authority’s lack of responsiveness to maintenance requests, “they kind of abandoned [us],” while another Hispanic older adult woman noted, “they already stopped fixing things. They used to come in quickly, but right now, I believe they already stopped.” Others complained about the incompetence or inadequate training of the maintenance staff, like this young adult Hispanic woman who said that the housing authority was “sending people that don’t even, you know, qualify for the job at all.”

Some residents responded to the unreliable maintenance by taking matters into their own hands, completing repairs themselves or hiring private contractors. As one middle-aged, Hispanic female participant relayed:

The adaptation I do is to try to tidy it up, to clean it, if there’s a little scratch, if there’s a little hole, to fill that hole and paint it, to keep it clean, to put things so that the insects, the cockroaches don’t get in…regarding maintenance, you can call them for some problem you have and they spend days and days and days and days and even months and no, they don’t fix it. In fact, many times I have had to choose to pay for [a private contractor] to do it because it bothers me.

Pests and rodents, including cockroaches, mice, and rats, were another common concern. As one middle-aged Black male resident noted:

I got big-ass bugs, just coming out whenever they want. And then when I get up in the middle of the night, I look at it, it looks at me, and…I just get my can of Raid and when I see them we have a little shootout < laughs>.

A middle-aged Asian female resident similarly relayed:

I sometimes see some rats here. When I see it, I go < repulsed sound>, I don’t know how to describe my feelings about it. But it’s not nice… Sometimes, there are quite a bit cockroaches at night. You cannot see them now, as they are all hiding…. Actually, you can also see a cockroach over there < laughing, points to a nearby cockroach>.

As these quotes illustrate, some participants expressed their sense of powerlessness and futility regarding their housing conditions by using humor or simply acknowledging their impotence in creating change. As the woman above continued, “Sometimes, I ask them to come over to repair something, such as replacing the stove, but no one comes. So, I have to give up. I just let it go.” In other instances, participants relayed how they adapted or took matters into their own hands to address their housing conditions; as she continued, “There was a leak in the toilet. So, I called them for help, but they didn’t come, so I stopped asking them for help. I took care of it myself.” Similarly, some residents described their efforts to focus on what they could control; as one young multiracial woman relayed her mother’s message, “The outside might not look the best but at least we can make, like, the inside look good.” Others took a more politicized view of their relative power, like a young Hispanic female participant who noted, “There are people who…if they have problems or something, say no, you can’t go to the [housing authority] office, or you can’t complain because you live in projects. But I say, yes, we live in projects, but we are human beings.”

A fourth arena of poor housing conditions was external to individual apartments, reflecting unsafe and unsanitary conditions in the building stairwells. Some residents complained of loud noises or smoke from neighboring apartments or hallways, and many others noted rats or mice in basements or stairwells, with one White older adult woman describing, for example, how “we was scared to go out in the hallway to get the mail because [my neighbor] said he’s seen two rats out there.” Broken stairs and the lack of elevators were also mentioned by numerous residents, serving as particular challenges for older residents with limited mobility. But by the far the most common concerns in this arena related to squatters living in and using drugs in building stairways, typically people experiencing homelessness and drug users from the nearby open-air drug market which the city was trying to shut down. Residents noted pervasive concerns over feces and urine, personal belongings, drug paraphernalia, and overdoses in the hallways. As one middle-aged Black woman noted:

The addicts are hanging in the hallway. I just went out before you came and a man was sitting right there in the wheelchair, see that wheelchair in the hallway? And he’s sitting out there shooting up, and I been calling ever since back in December for them to fix the door…because they broke the locks so they can come and go when they want. They use this hallway as a get-high–sleep hallway. ‘Cause before, back in December when I was complaining, you know, they had a ‘frigerator [on the landing between apartments]. They had a queen-size mattress cover.

A middle-aged Black male resident stated:

You’ve got your junkies coming into the buildings, however they’re getting into the building, whether the doors are left open, somebody opening it…. They’re upstairs in the hallways, got little cots, sleeping on cardboard boxes. You see needles everywhere.

While the vast majority of residents expressed deep and pervasive concerns about their housing conditions, a smaller number expressed satisfaction, like one Hispanic young adult male who said his apartment “has everything that we need.” Satisfaction with housing conditions was more common among residents with histories of housing precarity and residents of the limited number of townhouses. Townhome residents highlighted benefits such as outdoor space, greater privacy and autonomy, and more indoor space that was better maintained. As one middle-aged Hispanic woman stated:

At first I didn’t like it because I lived in the penthouse they call the fourth floor [top floor of apartment building]. I used to look at the roof. But after 17 years, I won the little house [townhouse] and I love it…it has three floors. It’s quiet.

In sum, concerns related to housing *conditions* were a pervasive stressor for participants, superseding, and often intersecting with, the other Cs of *cost*, *consistency*, and *context*.

### Consistency

*Consistency* is defined in the 4Cs framework as residential stability, encompassing a resident’s ability to remain in her home and the absence of threats of homelessness and displacement, overcrowding, and serial or forced moves (Swope & Hernández, 2019). Many of the participants in our sample had a uniquely high degree of residential instability prior to moving into their current apartments, which shaped their current experiences.

An overwhelming three quarters of the participants in our sample endorsed a history of housing inconsistency, with almost half reporting that they came to their current apartment from a shelter or homelessness. The uniquely high rate of experiences of homelessness may be driven by the housing authority’s policies which give housing applicants who are homeless priority for supportive housing, which is a common prioritization policy (Boston Housing Authority, 2025b; Rita et al., 2023). More than half of participants had experienced involuntary or serial forced moves, and about one third had experienced doubling up and overcrowding (including some who reported current doubling up). Further, more than one third of participants described a trauma history related to residential instability, such as domestic violence leading to a forced move or chronic health conditions or substance use disorders leading to homelessness.

With these experiences of instability as the backdrop, a primary theme related to housing *consistency* was, for many participants, appreciation for the roof over their heads and the stability that public housing had provided. Numerous residents noted that their move to public housing was a pivotal turning point in their lives. For example, one older White male participant who described a personal journey marked by struggles with substance abuse, complex medical problems, and homelessness told us about the moment his application for an apartment was accepted:

So, long story short, after a couple years, they sent me a letter [saying] we have found you an apartment…. So I cried and was like, give me that. And I came here. I was so happy. Yeah. Happy tears. So, I’ve been here since, uh, I signed the lease on [participant stated the exact date 20 years prior].

Another young, multiracial woman whose chronic illnesses had left her financially unstable and homeless, said, “coming from where I was coming from, it was a relief to have a place that was mine, not shared, and clean and decent.”

Perhaps because public housing represented a move toward consistency for so many participants, they were hyper-aware of threats to stability. More than half of participants described a looming fear of inconsistency, often related to the impending redevelopment of the community, and also to a general feeling of uncertainty and fear that any wrong move might land them back on the streets. Participants reported a broad sense of distrust, leaving them fearful of even run-of-the-mill interactions with the housing authority because of prior experiences or rumors about people being displaced, exemplified in stories like this one told by a middle-aged White woman:

Everybody gets anxiety about [the housing authority] people, workers coming in and having to go up to that office. Even my old neighbor, she got nothing to hide, but she’s like literally shaking at the thought of having to go up there. Everybody’s worried they’re gonna get thrown out…I’ve seen a few people that lived here their whole lives get kicked out…they had to get put in a shelter with their kids. And then like, reapply and do it all over again. So, that’s terrifying.

Participants specifying the redevelopment as the source of their distrust discussed how the threat of displacement hung over their heads. They expressed uncertainty about whether and when the redevelopment or another forced move might take place and reported that this uncertainty took a toll on their well-being. With more than half of the sample enduring forced moves in the past (often across public housing developments), feelings of uncertainty pervaded, as described by this middle-aged Hispanic woman:

I’ve already gone through a process where I lived in a project and [was] moved here. I know it’s not easy; there are many days of anxiety because you don’t know where they will take you, you don’t know if you’ll go back or what will happen. When it will start, and you have to start thinking about moving, where you will put your things, what will happen to you…this is not a game. I experienced it, and I know how difficult it is. When the last moment arrives, you have such anxiety that you want to die because you think you’ll be left homeless, that you won’t have anywhere to live.

Another young adult Hispanic woman reinforced how feelings of fear and uncertainty commingle and create anger:

Everyone is waiting on pins and needles to figure out what are the next steps, how their Christmas is gonna end up, how their summer is gonna end up. Where are they gonna end up?…I’ve been here, I earned my right. I’ve been here for 12 years. You know, I’ve never been a problematic [tenant].

She went on to describe how the uncertainty makes her feel, suggesting, like many other participants, that it strikes at her dignity, saying, “I don’t matter…. And that’s just, it just seems so cold. And as a community…we deserve better than that…it just scares me how, you know, I have my kids and…it’s just really gonna shake the community.”

Participants generally distrusted the information that they had received related to the redevelopment and, despite promises that new apartments will be reserved for all current residents, they worried. For example, one young Asian woman stated her concerns regarding the intersection of *consistency* and *cost*:

What I worry about is just not having enough homes for the low-income people. Because…like a lot of apartments that are going in now, yeah, like, they’re more for people that have money. That can pay the $1,000, $2,000 rent a month…I’m just worried that like, oh, you know, they’re gonna choose someone with more money than someone [like] my family.

In summary, participants’ histories of residential instability informed the ways in which they interpret threats to housing *consistency*, including the impending redevelopment of the community as well as run-of-the-mill interactions with the housing authority. Though many appreciated having a “roof over their heads” in light of histories fraught with housing inconsistency, an undercurrent of fear and distrust loomed large.

## Context

*Context* refers to broader neighborhood characteristics and resources in the 4Cs model, which is conceptualized to include institutions, social characteristics, walkability, green spaces, and other elements of the surrounding neighborhood (Swope & Hernández, 2019). In our interviews, most participants endorsed mixed views of the neighborhood context; as noted by one older White male participant, “It’s got its nice parts, but it also has its problems.” Regarding the positive aspects of context, the majority of participants appreciated neighborhood amenities, particularly highlighting walkability, public transit access, and institutional resources. For example, one older Asian woman described the neighborhood’s assets with pride, stating, “I’m proud I live here…it is very convenient. If I want to go to the [mall], I just walk…. Or I want to go along [to the] Vietnamese store, I just walk.” Another younger White woman noted, “there is so much going on. I like that it’s busy. I like having tons of restaurants, tons of people around. I like having connections…I like city life.” Many noted that the neighborhood was, as one young Hispanic woman put it, “very comfortable, a very good location.”

In addition to the convenience, many participants described how the close proximity to green space and beaches had a positive influence on their well-being. Many participants described walking along the nearby beach as part of their routine to combat stress. For example, one middle-aged Asian woman noted, “sometimes when I am in a bad mood, I just walk for a while…I just like walking here, walking by the sea.” Another participant, a middle-aged Hispanic woman, noted (with perhaps a touch of surprise) that neighborhood children have a walkable and safe atmosphere, saying, “kids can, like, even walk by themselves from a school bus drop-off or from school. I’ve seen some kids walking from…the bus to the house and there hasn’t been any kind of problem that I know of.”

In addition to structural resources, many participants also highlighted social resources. Nearly all participants endorsed positive or neutral relationships with their neighbors, shaping a neighborly and, in many cases, supportive community environment. Those that expressed neutral views on neighborliness generally noted that though their relationships were not deep, if help was needed people would be there. For example, one young Hispanic woman stated:

I think, everyone kind of just, I wouldn’t say minds their own business, but kind of, you know, when you see people you just say hi, but then they go about their way. I feel like though, like if people need help with something, depending on your relationship with the neighbor, like, they’ll help you out.

Others endorsed deeper supportive relationships, which included neighborly actions like picking up neighbors’ children from school and sharing meals. For example, a middle-aged Asian woman highlighted supportive neighbors who have a garden in the yard of their townhome that acts to both beautify the neighborhood and provide fresh food, saying, “It’s so close. We all know each other…. [My neighbor] gave me vegetables and flowers that we didn’t have. It’s good, I feel very friendly.” Overall, the appreciation for the neighborhood context prevalent in the interviews can be seen in this quote from a Hispanic Black middle-aged adult:

To me, it’s comfortable living here…the school for the little one is close by, the stores are close, the bus stop I use to go to work is close by. I can’t describe [the neighborhood] any other way than the best, the most comfortable, and ideal.

Despite these positive aspects of the neighborhood context, many participants’ views of the neighborhood were overshadowed by negative experiences. Pervasive issues with drug use, homelessness, and associated chaos permeated participants’ descriptions of both the conditions within their buildings and the neighborhood context. For instance, one young multiracial woman expressed multifaceted challenges and traumatic experiences in the neighborhood leading her to feel fearful:

Safety is such a big issue here… I don’t wanna get stabbed by a needle walking my dog. I don’t wanna have to fight with a drug addict because he wants me to give him twenty bucks to go get a score. I don’t wanna get robbed…I’ve been robbed twice in my life. I’ve been sexually assaulted, I’ve been attacked throughout these projects my whole life. I just need safety.

Though some participants reported feeling safe, most reported that they had witnessed or experienced violence and disorder in the neighborhood. Parents, in particular, expressed concern about neighborhood chaos and violence and its impact on their children’s well-being. One young Hispanic mother explained how the context made her hypervigilant:

We are unable to let our kids even go to the corner. My son is 12, never went to the corner store by himself. My [children] were never left outside by themselves without someone present or someone at the window vigilantly.

In addition to the general fear of violence and stress that comes with such vigilance, many participants shared personal experiences of witnessing or being victims themselves of violence. For example, one young Black mother shared that “a young boy got killed…within the first month of me living here. So that was kinda, like, shocking. ‘Cause I left [another housing project] due to a lot of shooting and my son being traumatized.”

The participants in our sample demonstrated a mixed perspective on neighborhood context, appreciating its convenience and sense of neighborliness while also expressing deep safety concerns. Some reported that the positive aspects helped them tolerate the negative aspects of the neighborhood, saying that things like the beach, parks, and green spaces “makes you forget the other part of the story” (middle-aged Hispanic woman). Others, like the middle-aged White man quoted below, took a resigned perspective, similar to responses to concerns with *conditions* and *costs*:

We’ve had people killed on the street too. Multiple people at that, in certain shootings. So that’s why we tend to just kind of mind our own business…we used to go out to school in the morning and were literally stepping over bodies of sleeping junkies and stuff. ‘Cause a lot of drugs are sold here…. But we just live our life, you know, it’s not gonna affect us either way. It is what it is. We are stuck here. We have to make the best of it, so that’s what we do.

## Crosscutting Themes: How the 4Cs Intersect

As delineated above, our findings suggest that experiences and tradeoffs across the 4Cs come together, for many, to form an overarchingly stressful residential environment with concerns about *costs*, *conditions*, *consistency*, and *context*. In the prior section, we discussed each of the 4Cs in turn, highlighting how they act independently and briefly noting intersections. Next, we expand upon the most salient intersections between the 4Cs, as elucidated by residents. We begin with *conditions*, and how responses to *conditions* are strongly shaped by intersections with the other domains. Then, we describe how threats to *consistency* powerfully interact with the other domains.

Residents’ responses to *conditions* were shaped by their intersections with *costs*, *consistency*, and *context*. Poor *conditions* were seen, in most cases, as a necessary tradeoff for relatively affordable *cost*, residential *consistency*, and retaining access to *context*ual amenities and kinship networks. In terms of the intersections between *conditions* and *cost*, residents described sub-par conditions but reported that they had no other options within their price range; as one young Hispanic woman noted after describing extensive issues with poor conditions and a lack of maintenance: “I don’t have nowhere else to go. So, I’m stuck…I don’t feel safe, I don’t feel comfortable…but what’s the alternative? Homelessness.” Further, many described perceived contradictions on the part of the housing authority, noting that they are slow to respond to maintenance concerns but quick to respond to rent violations; as this middle-aged Black Hispanic woman noted:

There are a couple of things in this house that are damaged and they’ve already been [reported to maintenance]. And they don’t respond to that. But try being late with the rent, you’ll see how they send you to court or kick you out. Yes, what they keep an eye on is the check.

These feelings of powerlessness to control *conditions* or leave for a better apartment, paired with distrust of the housing authority (driven in part by the housing authority’s perceived unresponsiveness but also by residents’ own histories of housing precarity) shaped another core intersection—between *conditions* and *consistency*.

Perceptions of *consistency* appeared to be the top priority and concern for many residents, shaping their responses to *conditions* as well as *cost* and *context*. For residents who had histories of homelessness, their perceptions of apartment *conditions* were relative to the conditions of life on the streets, so they were perhaps more willing to overlook *conditions*-related concerns than their counterparts. One formerly homeless resident, a White older adult, said most people would see the public housing development and his apartment as an “eyesore” that “looks ugly” but qualified these statements by saying “unless, of course, you’re coming from where I came from 20 years ago, which is the streets.” Another younger White woman who had come from homelessness said that, in most cases, she overlooked the lack of responsiveness by the housing authority: “There’s not always a fast response getting things fixed, which I don’t truly mind unless it has to do with my safety. Like, the front door won’t lock, so that can be anxiety inducing.” She went on to say that her past homelessness left her with debilitating anxiety and health issues, which at times were triggered by safety concerns in her building. She dealt with these issues by building relationships with neighbors and looking out for one another, in the absence of housing authority supports.

Residents also responded to *conditions*-related concerns in ways that they hoped would preserve their *consistency*. For example, one middle-aged Black woman described how she responded to poor conditions by withholding rent, but then described how she feared losing this potential leverage in the face of redevelopment, surmising that withholding rent may now be used against her when it comes to relocation, saying, “[poor housing conditions are] a violation, and they wonder why sometimes I hold my rent. I have a right to, I know what my lease tells me. But…I hope that don’t stop me from getting an apartment.” Other residents shared humiliating experiences with housing authority staff and/or histories of trauma that led them to limit their interactions with any perceived authority figures, together compounding concerns that interactions with the housing authority could lead to eviction (in*consistency*). For example, one multiracial young adult resident shared that:

[I’ve been] cussed out and yelled at by management. I just wasn’t respected by them at all. I had a manager come in, I was a child, and they started taking pictures [of a pest infestation in her home]… managers and [maintenance men] started coming and they started taking their personal phones and taking pictures <participant mimics people pointing and laughing>. And they tried to get us evicted. And I like, have PTSD from that. They also like to show up with no notice…. They’re like, we have to come in. They give you no options. You feel like they’re above you. Like they’re running your life…I literally do freak out [when they come]…they just need to understand that these are our lives they are playing with…if I have no house, I lose my [child]. I most likely would go off the deep end.

While her experience with housing authority staff was more emotionally damaging than most, distrust of the housing authority and fear of retribution were common crosscutting themes, leaving residents to deal with poor *conditions* on their own to retain *consistency* in their homes.

The *conditions–cost* intersection extended, for many residents, to *context*. The key tradeoff noted between *conditions* and *context* was that residents reported that they had to trade poor *conditions* within their apartments and apartment buildings to retain broader neighborhood *context*ual amenities. A middle-aged Black mother described the tradeoffs this way: “I can’t fix a lot of things…as a mom living in this project, I always live with that tension, because I don’t want to lose my house, I can’t pay normal rent and we have to keep on our toes.” While residents endorsed a mixed view of the neighborhood, most commended the neighborhood’s rich amenities and their kinship networks and could not retain those on the private market due to *cost* constraints. Participants reported deep roots and place attachment in the neighborhood saying “I really do love [the neighborhood]” and “I feel a lot connected to the neighborhood, I have my roots here…I feel like I belong to [the neighborhood].” One middle-aged White mother noted issues with run-down building conditions and drug use, but said that if the conditions were improved, she wished to stay in her affordable unit in the neighborhood, “because you know what? I don’t think I could afford anywhere else in this town and it, I really do love [this neighborhood].”

The intersection between *consistency* and *context* was particularly strong in our data. Residents craved consistency, in part due to the residential histories described previously but also because their lives had become rooted in the neighborhood. Residents’ fears were amplified by the impending community redevelopment—which enhanced their distress about increasing *costs* and loss of *context*, the loss of both local amenities and social ties. Uncertainty about what would come next permeated their descriptions, exemplified by this Hispanic young woman’s summary:

We have a life here. My kids go to school here…I’ve been here in [this neighborhood] all my life…I’m gonna have to, you know, change services…go to a whole different place to shop, go to a whole different place for my healthcare…the future looks bleak…God be with us because at this point, everything here is unsure.

While the neighborhood context, for many, was among the bright spots in their descriptions, a key fear was that if *costs* increased or redevelopment led to displacement, they would be unable to remain in the neighborhood and sustain access to its many resources and their social connections. One older Hispanic Black woman described friendly neighbors and instrumental support, saying, “this neighborhood is amazing, I love it here,” but went on to express fear that the transition to mixed-income development will mean displacement of low-income residents, saying:

What will happen with all the people who don’t have the good income? I heard people say people making more money, they will be able to move in. What about people who don’t make that much money? Who are already here? I wish I knew.

Another resident described her anxiety over the impending redevelopment and losing her community ties, saying, “I’ve lived in [this neighborhood] for 23 years now. Telling me I’m going to go to [another neighborhood] is like starting all over again.”

For many participants with negative experiences across the 4Cs, there was a notable tone of apathy and hopelessness. These residents recognized that, though they could control some conditions within their own apartments, more serious housing conditions and building and neighborhood contexts, as well as the ability to move elsewhere, were largely out of their control. One particularly demoralized participant, a young Hispanic mother, described how experiencing insults across the 4Cs congealed into deep hopelessness:

It’s a bad area. It’s literally where problems and problematic people congregate…there’s not very many opportunities for bettering…and there’s no way to progress…as soon as I start working, my benefits go down. My rent goes higher…. So as much as I work, I’m basically working for nothing. And how am I supposed to get out the projects?… I have a psychologist for everything because, you know my house supposed to be my domain. It’s supposed to be…my sanctuary, where I feel safe… The problems, the plumbing, the noises, the problems you have to step outside the door and seeing like, you know, those big roaches just dead and, cigarette smells and bottles and needles and just like, it makes me just wanna stay in bed. Because anything outside my bed is just chaotic. It’s very stressful. Very stressful. It makes you mad. It makes you just want to give up. You know what I mean? … So, you feel hopeless. So, you feel like giving up. And that’s just the attitude everywhere here.

In sum, distrust, in some cases rooted in residents’ histories of housing insecurity and trauma, amplified concerns surrounding each of the 4Cs and their intersections, and reinforced perceived stressors.

## Discussion

In this article, we drew on the perspectives of residents of a large urban public housing development to further elucidate the 4Cs model of housing *cost*, *conditions*, *consistency*, and *context*, and to address gaps in the literature on the experiences of low-income renters in contemporary public housing. This research is situated in a changing urban environment, within a racially and ethnically diverse public housing community surrounded by a gentrifying and highly resourced community within a time of exploding housing unaffordability and precarity for low-income Americans. Moreover, reflecting federal and local policy initiatives, this work assesses a community on the cusp of a comprehensive shift through the phased demolition and redevelopment of their public housing development into a mixed-income community. As such, our results on the experiences and intersections among the 4Cs adds to existing research by assessing a relevant new context that differs from that of prior evidence, much of which was derived from more racially and economically segregated public housing communities and assessed the repercussions of earlier (now outdated) redevelopment models.

Some prior research has found low-income public housing residents to be better off than those in other low-income rental situations, particularly in the arenas of *cost* and *consistency*, reflecting affordable housing policies supporting rent caps and other tenant protections (DeLuca & Rosen, 2022; DeVoss et al., 2022). However, although some positive experiences and perceptions emerged, many public housing residents in our sample described challenges across the 4Cs, situating the 4Cs components as intersecting and mutually reinforcing. They often reported accepting significant insults in some areas (namely, *conditions*) to retain benefits in others, including *cost*, *consistency* and, in a more mixed fashion, *context*. Yet, they also expressed notable worries about the upcoming redevelopment interrupting their housing *consistency* and capped *costs*, with these worries far outstripping any expressed excitement about improved housing *conditions* and neighborhood *context* promised in the redevelopment plans. Indeed, some of the participants in this study relayed a pronounced sense of hopelessness resulting from accumulated challenges across the 4Cs. These findings advance knowledge by providing new empirical evidence from a diverse sample of public housing residents from one of the many public housing communities that was not targeted by large-scale public housing redevelopment efforts like HOPE VI and Choice Neighborhoods. New models of public housing redevelopment are explicitly designed to limit displacement, sustain resident social connections, and engage residents in redevelopment planning. Yet our evidence on the experiences of residents in a community embarking on this new model of redevelopment highlights continued concerns related to displacement and change, amplified by distrust of the housing authority. They further offer insights into how new policies prioritizing people who have experienced homelessness for public housing units may affect resident experiences and perceptions of public housing communities. These results thus have implications for future redevelopment efforts and community building initiatives. The following section will describe how our findings enhance empirical evidence on how the 4Cs act independently and collectively.

In terms of *cost*, our findings suggest that, while some residents expressed appreciation for the fixed-cost formula used to calculate rent in public housing and appreciated that private housing options were wholly unaffordable and out of reach, many nonetheless strained to make ends meet. Some residents reported that the cost formula, wherein their rent increases with increases in income, sometimes enforced unwanted *consistency* by limiting their opportunities for advancement out of public housing, and intersected with concerns over *conditions* and *context*. Further, a smaller but vocal subset of our sample described *cost*-related resentments, describing high rents for substandard conditions and cost disparities between tenants driving bitterness and frustration, while others expressed distrust of the promised flexibility wherein rent should decrease in response to a loss of income. These results further contour prior literature exploring affordability in public housing, which has primarily focused on the protections offered by the tenant payment structure with less exploration into the possible drawbacks of the formula and intersections with other dimensions of the 4Cs (DeVoss et al., 2022).

Prior literature on *conditions* in public housing has frequently aggregated varying aspects of conditions (e.g., pests, maintenance problems), and, in many cases, has focused on the experiences of movers (e.g., those who are displaced as a result of public housing redevelopment, or comparing housing conditions pre-and-post eviction) (DeVoss et al., 2022; Schmidt, 2024). Our results replicate these findings in a sample of contemporary public housing residents remaining in place. Our findings suggest that, in this sample, deficient and in some cases hazardous housing conditions are experienced as significant, near-constant stressors. Though residents describe the conditions as substandard, they also describe how they make concessions (e.g., DIY repairs, avoiding calling for assistance) because they perceive themselves to be powerless to enact longer-term solutions to these substandard conditions, and fear potential repercussions in relation to *consistency*. Our findings add to a small but growing body of qualitative literature that aims to elucidate the experiences of housing conditions among low-income renters in today’s context of severe housing shortages and gentrifying urban contexts which further constrain the housing options of low-income renters. For example, Marquez et al. (2019) found, among a sample of low-income Nevada renters, feelings of powerlessness over participants’ poor housing conditions and that landlords’ “Mickey Mouse” solutions to housing problems led to pervasive concerns, but these concerns were seen as tradeoffs for maintaining *cost* and *consistency*. Further, our findings lend support for Schmidt’s (2024) conceptualization of how low-income renters engage in a complex dance that he calls “negotiating neglect,” in which they weigh whether it is worth notifying landlords of maintenance problems and enduring repair delays (and possible retribution) or whether to accept substandard conditions because there are few alternatives.

Much prior research on low-income renters has focused on experiences of housing instability and the benefits of *consistency* (Schmidt, 2024). Our findings in this domain nuance this literature. For many in our sample, the move to public housing was a pivotal event, offering consistency after traumatic histories of housing instability. As demand for affordable housing continues to grossly exceed supply, policies prioritizing individuals experiencing homelessness are common in municipalities across the country (Rita et al., 2023). Our results highlight how resultant changes in the population of public housing may alter residents’ perceptions of their housing contexts and responses to housing authority policies. Beyond the gratitude for consistency expressed by many in our sample, however, perceived precarity, perhaps rooted in trauma histories, often remained, with residents relaying worries related to the impending redevelopment including fears of displacement, their ability to remain in the broader neighborhood, and cost increases. These fears were particularly salient in a context of neighborhood gentrification in a city with highly elevated housing costs, which for many participants cast a shadow over the hoped-for benefits of redevelopment. Even with substantial efforts by the housing development company to share information and solicit resident feedback on redevelopment plans, our results extend the limited recent work on residents in contemporary public housing facing redevelopment who describe the impending redevelopment as an emotional and anxiety-provoking stressor, making it difficult to plan ahead and symbolizing their powerlessness against forced relocation (Watt, 2023; Westbrook et al., 2024).

Though there is a relatively robust literature on neighborhood *contexts* in public housing communities, prior literature has largely focused on public housing neighborhoods as sites of concentrated disadvantage, with a focus on contexts characterized by violence and disorder and much less attention to contextual amenities (DeVoss et al., 2022; Nebbitt, 2015; though see Coley et al., 1997, 2023). To be sure, participants in our study endorsed a broad array of contextual stressors, primarily related to drug use and violence, and to the recent surge of outsiders experiencing homelessness squatting in building hallways and landings. Yet in many of our interviews, descriptions of the neighborhood context provided a bright spot, with neighborly relationships and local amenities buffering concerns in other arenas, which reflects forms of community cultural wealth (Wang et al., 2026). However, these bright spots intersected with *cost* and *consistency* anxieties concerning whether they would be able to remain in the neighborhood post-redevelopment due to increased costs, as well as a lack of other affordable options if they were displaced. Like other recent studies of low-income renters, the challenges of the increasingly unaffordable housing market, felt particularly in gentrifying urban areas, can leave low-income renters feeling stuck or powerless, enduring substandard *conditions* to attain *context*ual amenities, *consistency*, and affordable *costs* (Marquez et al., 2019; Rosen et al., 2023; Schmidt, 2024). Indeed, a recent national report found that 83% of low-income households live in unaffordable housing (Airgood-Obrycki et al., 2024), reinforcing our participants’ concerns that alternate options are not accessible.

Our finding that some participants see themselves as accepting deficient housing *conditions* for access to *context*ual amenities diverges in some ways from past scholarship on public housing residents relocating using housing vouchers, which found that low-income movers prioritized housing unit quality over neighborhood context (Rosenblatt & Deluca, 2012). Residents in our sample often described deep appreciation for neighborhood amenities from social networks to nearby beaches, public transit, and services, and the ways in which these relationships and amenities fostered their attachment to the neighborhood. They further, notwithstanding the racial and ethnic diversity of the community, generally described friendly or at least neutral social connections and a shared sense of community. And numerous residents, reflecting on histories of homelessness and trauma, saw relative safety and calm within their public housing context. These unique findings provide new empirical evidence that residents of large public housing developments can identify tangible benefits from well-resourced communities, even prior to public housing redevelopment and investments, and suggest important targets for redevelopment initiatives seeking to both sustain and increase economic and social opportunities for public housing residents.

Together, our results suggest numerous areas of intersection among the 4Cs of housing, with residents facing tradeoffs and seeing both strengths and challenges across domains. Our findings suggest that each of the 4Cs cannot be viewed in a vacuum and they should be seen as acting interactively. Overall, we find that residents endure poor *conditions* and view these as a necessary tradeoff to maintain *consistency*, because moves are *cost* prohibitive and may require moving outside the neighborhood, losing the amenities and social connections they value in the *context*. Powerlessness and distrust were key crosscutting themes as residents described their experiences of the 4Cs, with the impending redevelopment increasing fears of losing *consistency* and facing increased *costs* that overshadowed the hoped-for improvements in *context* and *conditions*.

## Policy Implications and Conclusions

Our findings point to important policy and practice implications, and raise questions about current approaches to public housing redevelopment. Earlier models of public housing redevelopment imposed massive displacement, with displaced residents often experiencing increasing inconsistency and poor conditions and contexts (Aratani et al., 2019; Keene & Geronimus, 2011; Keene & Ruel, 2013). Seeking to learn from the lessons of prior efforts, new models of redevelopment seek to promote *consistency* (limiting displacement and promising new units to all current residents) while grossly improving housing *conditions* and *contexts* and retaining affordable *costs*. Although the developer in our study community has undertaken extensive community engagement efforts regarding the planned redevelopment, numerous residents expressed fear and stress related to potential displacement and the loss of *consistency* and access to community amenities, while others relayed concerns regarding increased *costs*. These results provide early evidence on new redevelopment approaches and suggest that despite noteworthy communication and engagement efforts, currents of distrust remain prevalent. Policies and practices related to public housing redevelopment might benefit from the use of trauma-informed community building strategies (e.g., build trust and transparency, acknowledge historical trauma) designed to more deeply engage with residents, limit misinformation, and ease redevelopment-related stress (Falkenburger et al., 2018). This could ease fears of in*consistency* and *cost*-related concerns.

We found that *conditions* were experienced as gravely substandard, with some participants going so far as to characterize the conditions as a violation of their human rights. Though the redevelopment of this public housing community will bring with it vastly improved housing *conditions*, residents may not see these improvements for many years. In the meantime, our findings call for immediate attention to health and safety concerns, as residents largely feel powerless to enact change amidst the crumbling physical infrastructure. Trauma-informed community building approaches may be useful here as well, providing transparency and follow-through in relation to maintenance requests. Approaches that connect various city systems such as 311, public health surveillance systems, and inspectional services may help address pressing health and safety concerns. It is essential to highlight that in the midst of our data collection, the housing authority launched a comprehensive assessment of policies and practices related to maintenance conditions (Bok, 2025). As a result, starting in 2023 the agency began implementing a multi-pronged maintenance systems change, including more inspections and improved staffing. Following the completion of our data collection, they digitized their work order system and began addressing the backlog, with a report from the housing authority indicating an 83% decline in open maintenance orders by March 2025 (Bok, 2025). It is vital to track the effects of this policy shift on residents’ perceptions of housing conditions, as well as of the redevelopment process, which was launched in June 2025; these are core goals of future waves of the HOME Study.

Our findings also hold policy implications in relation to *costs*. Though public housing residents are ostensibly protected by a fixed rent calculation formula, many pointed to concerns with the formula, namely that it acted to limit upward mobility and disincentivize work. Navigating household economic decisions when an increase in income or an improvement in job status may lead to a reduction in public benefits and increased rent left residents in our sample lamenting a system that made them feel trapped. There is bipartisan concern regarding policies that may disincentivize work, and prior literature has suggested a number of policy avenues for alleviating disincentives driven by benefits cliffs, wherein sudden drop-offs in benefits occur at certain income thresholds (Ballentine et al., 2022). Such suggestions may also be applicable to the public housing rent formula: incorporating more flexibility in the rent calculation as resident incomes increase or routing portions of increased earnings to savings for downpayment costs could support resident financial stability and promote economic mobility. Federal and local efforts have seen promise in interventions like temporary payments to make up for lost benefits or more gradual reductions in benefits (versus strict income thresholds), and some more conservative approaches call for shifting from the current 30% of household income on rent formula to a flat rent rate (Encouraging Upward Mobility, 2025). Further research is needed to elucidate alternative approaches that support both resident stability and economic mobility.

In terms of *context*, residents highlighted concerns regarding drug use, homelessness, and violence (primarily from individuals coming from outside the community), but also appreciated the neighborhood amenities and their kin-like social networks. Research suggests that prior public housing redevelopment efforts with large-scale displacement were perceived by residents as having improved aspects of *context* such as safety, but that those improvements were counterbalanced by the devastation of social networks caused by relocation (Aratani et al., 2019). Very little evidence exists on effects of newer models of phased redevelopment. While phased redevelopment approaches are expected to limit displacement and hence resultant disruptions to social networks and place attachment, prior research suggests that residents should be meaningfully engaged in efforts to improve community contexts in order for them to retain access to the benefits afforded by redeveloped communities (Chaskin & Joseph, 2019). Approaches such as “housing plus services” models that integrate case management, supportive services, and community capacity building may help residents take advantage of contextual amenities and simultaneously address concerns related to *cost, conditions*, and *consistency* (Foell, 2026).

Our study explored the housing and neighborhood experiences of a sample of adults from a single urban public housing development in Boston. Though these findings relate to a specific local context, embedded within a gentrifying broader neighborhood within a high-cost city, many components are likely transferable to other, similar public housing communities and low-income renter populations. Housing cost increases and limited supply are rapidly growing concerns, even among previously more affordable cities and rural areas across the U.S., with more than 4 in 5 low-income renters living in unaffordable housing (Airgood-Obrycki et al., 2024). This growing unaffordability suggests that aspects of the *cost* concerns our results highlighted—which led to notable stress and feelings of being “stuck”—are likely broadly shared. In contrast, national data indicate that 12% of low-income renters live in substandard quality units (Joint Center for Housing Studies, 2024), suggesting that the breadth and depth of poor housing conditions we unearthed may be less commonly shared. In considering generalizability and unique contributions, it is also important to reiterate the broad diversity of our sample across dimensions of age, race, ethnicity, language, and gender. As such, our results extend prior literature which has largely been conducted in more racially segregated public housing communities or focused on narrower populations of families with children (Popkin et al., 2009). Our research draws on these diverse perspectives but did not undertake a full comparative subgroup analysis, which could facilitate a more systematic assessment of similarities and differences across identities. Future research would benefit from such an analysis, which could pinpoint differential experiences and hence inform future programming and policy for affordable housing.

Our study results expand understanding of the unique strengths and stressors in the residential environment of contemporary public housing residents and the ways that they experience the 4Cs. We found that though public housing provides affordances in the way of *cost* and can provide much needed *consistency* for residents, and that some residents appreciated aspects of the *conditions* and *context*, there were overwhelming safety and health concerns, as well as notable *cost* and *consistency* worries, associated with psychological and physical stress and diminished well-being. These tradeoffs undermine the goals of the public housing program to provide decent, stable, affordable housing. Meaningfully incorporating resident voices in redevelopment processes, promoting transparency in redevelopment approaches and timelines, and retaining key social connections in the context of widely implemented, phased redevelopment processes holds promise for improving residents’ experiences.

## Figures and Tables

**Table 1.** Demographic characteristics of participants (*N* = 61).

Variables	*N*	Percent	Mean (*SD*^a^)	Range
Age (years)	61		45.64 (18.74)	18–92
Gender				
Female	49	80.33		
Male	12	19.67		
Race				
Non-Hispanic White	12	19.67		
Non-Hispanic Black	10	16.39		
Hispanic or Latino	24	39.34		
Asian	9	14.75		
Multiracial	6	9.84		
Language				
English	40	65.57		
Spanish	15	24.59		
Cantonese/Mandarin	6	9.84		
Home type				
Apartment building	39	63.93		
Town home	22	36.07		
Tenure (years)	59		15.56 (11.05)	0–46

a SD, standard deviation.
